# Sweet sensors support stressed cell survival

**DOI:** 10.1371/journal.pbio.3001705

**Published:** 2022-07-22

**Authors:** Nathaniel J. Himmel, Richard Benton

**Affiliations:** Center for Integrative Genomics, Faculty of Biology and Medicine, University of Lausanne, Lausanne, Switzerland

## Abstract

Gustatory receptors on sensory neurons have a well established role in chemosensation. This Primer explores the implications of a PLOS Biology study revealing that a gustatory receptor gene cluster has an unexpected role in regulating cell survival during proteotoxic stress.

## Teaser

Gustatory receptors (Grs) are well known for their functions in sensory neurons in detecting food and toxins. An intriguing new study in *PLOS Biology* provides evidence for a role for Grs in *Drosophila* epithelia in protecting stressed cells from proteotoxicity.

Animal olfactory and gustatory receptors serve as the molecular interface between the external chemical world and the nervous system. These receptor repertoires are typically large and divergent, and individual members are expressed in defined peripheral sensory neuron/cell populations where they detect specific chemical cues to evoke, ultimately, appropriate behaviors [1,2]. However, there is growing appreciation that chemosensory receptors have other (potential) functions beyond sensing environmental signals [3,4]. Some roles are essentially “chemosensory,” such as those of mammalian taste receptors in gut nutrient responses or olfactory receptors in sperm chemotaxis; other roles may reflect distinct cellular functions, including in muscle regeneration and adiposity [3,4].

**Fig 1:**
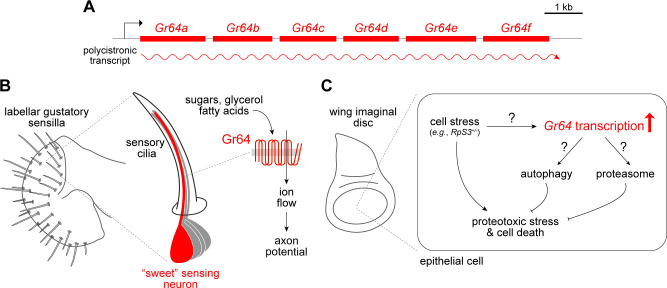
Tissue-specific roles for Gr64 receptors in taste sensing and cell stress sensing. (A) Schematic of the tandem cluster of *Gr64* genes, which are thought to be transcribed as a polycistronic transcript [9]. (B) Left: cartoon of the *Drosophila* feeding organ, the labellum, which is covered in gustatory sensilla housing different types of ciliated sensory neurons. Middle: *Gr64* genes are expressed, in various combinations, in typically a single “sweet”-sensing neuron in each sensillum. Right: individual Gr64 proteins contain 7 transmembrane domains and likely assemble into homo- or heteromeric complexes to form ligand-gated ion channels responding to appetitive chemicals, including many sugars, glycerol, and fatty acids [6]. (C) Model for the role of *Gr64* genes in promoting proteostasis and cell survival in imaginal discs under proteotoxic stress (see text); the mechanistic function of *Gr64* genes in this process is unclear, but is thought to be largely cell autonomous [5].

A Discovery Report in this issue of *PLOS Biology* from Baumgartner and Mastrogiannopoulos in the Piddini group [5] proposes a striking new role for the *Drosophila Gustatory receptor 64* (*Gr64*) gene cluster (Fig 1A) in stress responses of epithelial cells. This finding is surprising as the Gr64 receptors (hereafter, “Gr64s”) have been intensively studied in insects for their functions as ligand-gated ionotropic receptors for various sugars and other appetitive chemicals in specific populations of sensory neurons (Fig 1B) [6].

The study was motivated by a curious observation emerging from the group’s previous work investigating cell competition in wing imaginal disc epithelial cells [7]: Mutations that cause proteotoxic stress (either in a ribosome subunit gene (*RpS3*) or a E3 ubiquitin ligase gene) led to approximately 10-fold transcriptional up-regulation of the *Gr64* gene cluster. Such stressed cells are normally eliminated from the disc epithelium by apoptosis, particularly when neighbored by wild-type cells in mosaic tissue [7,8]. In the new work [5], the authors tested whether up-regulation of *Gr64* expression has a physiological significance in the process of stress-evoked cell elimination.

Indeed, loss of one copy of the *Gr64* cluster enhanced the frequency of death of stressed disc cells (*RpS3*^*+/*−^), compared to control, unstressed (*RpS3*^*+/+*^) tissue. This result implied a protective role for one or more of the Gr64s in promoting survival of stressed cells. The authors substantiate this conclusion using various individual *Gr64* gene loss-of-function alleles, RNA interference (RNAi), and transgenic rescue, although the polycistronic nature of *Gr64* transcripts (Fig 1A) [9] complicated determination of whether this pro-survival role requires specific *Gr64* genes or is a collective function of the entire cluster.

To understand how *Gr64* genes contribute to cell survival under stressed conditions, the authors restricted *Gr64* RNAi to the posterior compartment of the wing disc (in an *RpS3*^*+/*−^ background) that led to a milder phenotype, with low frequency of cell death, and compared the expression of various markers of cell stress in anterior (control) and posterior compartments. Knock-down of *Gr64*s led to modest increases in expression of a reporter for the oxidative stress response pathway (GstD1-GFP) and markers for the integrated stress response (phospho-JNK and phospho-eIF2α), consistent with the hypothesis that Gr64s function to attenuate cell stress.

In their previous work [8], the authors showed that stress in *RpS3*^*+/*−^ cells results from accumulation of protein aggregates, due to decreased protein degradation/removal through the proteasome and/or autophagy. In the new study, they probe the mechanistic basis of how Gr64s help promote cell survival using transgenic reporters for the activity of these pathways (Proteoflux for the proteasome and ReFlux for autophagy [8]). Loss of Gr64s reduced both proteasome and autophagosome function, consistent with a model in which these proteins help prevent proteotoxic stress.

This report provides evidence for a new role for Grs in a cell survival pathway (Fig 1C). As with all provocative discoveries, the work raises several important questions. First, how are *Gr64* genes transcriptionally up-regulated in response to cellular stress? The mechanism is likely to be distinct from the developmental pathways controlling the neuron-specific expression of Grs in chemosensory organs (Fig 1B) [6]. However, *Gr*s (and other chemosensory genes) display plasticity in expression levels in adults, suggesting that other global mechanisms of gene regulation exist. Stress-induced *Gr64* expression in wing discs was inferred from bulk RNA-sequencing, so it is unclear whether *Gr64* up-regulation depends upon an external signal acting across the tissue or occurs in a cell-autonomous manner. In situ analysis of *Gr64* expression is clearly warranted.

Second, are Gr64s functioning as ligand-gated channels in stressed cells? While it is reasonable to presume that they have a similar biochemical function to their role in chemosensory neurons, protein localization analyses will be essential to determine if Gr64s are present on the plasma membrane of epithelial cells (and therefore able to respond to extracellular cues) or restricted to intracellular membranes. In either scenario, known Gr64 ligands (sugars, glycerol, and fatty acids [6]) are likely to be available to gate these channels, although whether such chemicals could act as deterministic signals is unclear. Alternatively, Gr64s may function in a ligand-independent manner, conducting ions through spontaneous channel opening. It is notable that other (chemosensory) ionotropic receptors are up-regulated in stressed wing discs [7], raising the possibility that multiple ion channels participate in the proteostasis pathway.

Third, assuming Gr64s do function as channels, how might ion conduction suppress proteotoxic stress? The authors hint that Ca^2+^ is involved, through their observation of decreases in frequency of “calcium flashes” in disc cells when *Gr64*s are down-regulated. However, how Gr64s contribute to such flashes and how this phenomenon is related to regulation of the proteasome or autophagy are completely unknown. Of course, we cannot rule out that the Gr64s have a distinct function in this cellular context, unrelated to their signaling mechanism in sensory neurons.

Despite the abundance of questions, this intriguing work outlines another example of a non-chemosensory function of chemosensory receptors. This role is surprising in the context of our prior appreciation of Gr64s only as sweet sensors, but the Gr superfamily is evolutionarily ancient: Homologs are present in animals, plants, and unicellular eukaryotes, with some, albeit very sparse, evidence for non-chemosensory activities [10]. Further investigation of Gr64s’ contribution to protection of cells from stress could illuminate an ancestral function for this protein family.

## References

[1] Su CY, Menuz K, Carlson JR. Olfactory perception: receptors, cells, and circuits. Cell. 2009;139(1):45–59. doi: 10.1016/j.cell.2009.09.015 19804753 PMC2765334

[2] Yarmolinsky DA, Zuker CS, Ryba NJ. Common sense about taste: from mammals to insects. Cell. 2009;139(2):234–244. doi: 10.1016/j.cell.2009.10.001 19837029 PMC3936514

[3] Lee SJ, Depoortere I, Hatt H. Therapeutic potential of ectopic olfactory and taste receptors. Nat Rev Drug Discov. 2019;18(2):116–138. doi: 10.1038/s41573-018-0002-3 30504792

[4] Dalesio NM, Barreto Ortiz SF, Pluznick JL, Berkowitz DE. Olfactory, Taste, and Photo Sensory Receptors in Non-sensory Organs: It Just Makes Sense. Front Physiol. 2018;9:1673. doi: 10.3389/fphys.2018.01673 30542293 PMC6278613

[5] Baumgartner ME, Mastrogiannopoulos A, Kucinski I, Langton PF, Piddini E. The Gr64 cluster of gustatory receptors promotes survival and proteostasis of epithelial cells in *Drosophila*. PLoS Biol. 2022; 20(7):e3001710. doi: 10.1371/journal.pbio.3001710 35862315 PMC9302837

[6] Chen YD, Dahanukar A. Recent advances in the genetic basis of taste detection in *Drosophila*. Cell Mol Life Sci. 2020;77(6):1087–1101. doi: 10.1007/s00018-019-03320-0 31598735 PMC7125039

[7] Kucinski I, Dinan M, Kolahgar G, Piddini E. Chronic activation of JNK JAK/STAT and oxidative stress signalling causes the loser cell status. Nat Commun. 2017;8(1):136. doi: 10.1038/s41467-017-00145-y 28743877 PMC5526992

[8] Baumgartner ME, Dinan MP, Langton PF, Kucinski I, Piddini E. Proteotoxic stress is a driver of the loser status and cell competition. Nat Cell Biol. 2021;23(2):136–146. doi: 10.1038/s41556-020-00627-0 33495633 PMC7116823

[9] Slone J, Daniels J, Amrein H. Sugar receptors in *Drosophila*. Curr Biol. 2007;17(20):1809–1816. doi: 10.1016/j.cub.2007.09.027 17919910 PMC2078200

[10] Benton R, Dessimoz C, Moi D. A putative origin of the insect chemosensory receptor superfamily in the last common eukaryotic ancestor. Elife. 2020;9:e62507. doi: 10.7554/eLife.62507 33274716 PMC7746228

